# DNA methylation reveals distinct cells of origin for pancreatic neuroendocrine carcinomas and pancreatic neuroendocrine tumors

**DOI:** 10.1186/s13073-022-01018-w

**Published:** 2022-03-01

**Authors:** Tincy Simon, Pamela Riemer, Armin Jarosch, Katharina Detjen, Annunziata Di Domenico, Felix Bormann, Andrea Menne, Slim Khouja, Nanna Monjé, Liam H. Childs, Dido Lenze, Ulf Leser, Florian Rossner, Markus Morkel, Nils Blüthgen, Marianne Pavel, David Horst, David Capper, Ilaria Marinoni, Aurel Perren, Soulafa Mamlouk, Christine Sers

**Affiliations:** 1 Charité – Universitätsmedizin Berlin, corporate member of Freie Universität Berlin and Humboldt-Universität zu Berlin, Institute of Pathology, Charitéplatz 1, 10117 Berlin, Germany; 2 Charité – Universitätsmedizin Berlin, corporate member of Freie Universität Berlin and Humboldt-Universität zu Berlin, Hepatology and Gastroenterology, Berlin, Germany; 3 Institute of Pathology, University of Bern, Murtenstrasse 31, 3008 Bern, Switzerland; 4 Bioinformatics.Expert UG, Berlin, Germany; 5 Humboldt-Universität zu Berlin, Knowledge Management in Bioinformatics, Berlin, Germany; 6 Integrative Research Institute (IRI) Life Sciences, Humboldt-Universität zu Berlin, Berlin, Germany; 7 Charité – Universitätsmedizin Berlin, corporate member of Freie Universität Berlin and Humboldt-Universität zu Berlin, Institute of Neuropathology, Berlin, Germany; 8 German Cancer Consortium (DKTK); Partner Site Berlin, German Cancer Research Center (DKFZ), Heidelberg, Germany

**Keywords:** Cell-of-origin, Epigenetics, Pancreatic neuroendocrine neoplasm

## Abstract

**Background:**

Pancreatic neuroendocrine neoplasms (PanNENs) fall into two subclasses: the well-differentiated, low- to high-grade pancreatic neuroendocrine tumors (PanNETs), and the poorly-differentiated, high-grade pancreatic neuroendocrine carcinomas (PanNECs). While recent studies suggest an endocrine descent of PanNETs, the origin of PanNECs remains unknown.

**Methods:**

We performed DNA methylation analysis for 57 PanNEN samples and found that distinct methylation profiles separated PanNENs into two major groups, clearly distinguishing high-grade PanNECs from other PanNETs including high-grade NETG3. DNA alterations and immunohistochemistry of cell-type markers PDX1, ARX, and SOX9 were utilized to further characterize PanNECs and their cell of origin in the pancreas.

**Results:**

Phylo-epigenetic and cell-type signature features derived from alpha, beta, acinar, and ductal adult cells suggest an exocrine cell of origin for PanNECs, thus separating them in cell lineage from other PanNENs of endocrine origin.

**Conclusions:**

Our study provides a robust and clinically applicable method to clearly distinguish PanNECs from G3 PanNETs, improving patient stratification.

**Supplementary Information:**

The online version contains supplementary material available at 10.1186/s13073-022-01018-w.

## Background

Pancreatic neuroendocrine neoplasms (PanNENs) have undergone several classification changes according to consensus guidelines [1]. In 2017, these rare tumors, accounting for 2% of all pancreatic malignancies [2, 3], were grouped by the WHO with respect to proliferation index and morphology. With this approach, PanNENs fall into two basic subtypes: well-differentiated pancreatic neuroendocrine tumors (PanNETs) and poorly differentiated pancreatic neuroendocrine carcinomas (PanNECs) [4]. PanNETs are further divided into G1, G2, or G3 tumors, depending on their proliferation index (Ki67 <3%, 3–20%, and > 20%, respectively), with an increasingly malignant nature [4–6]. PanNECs are all high-grade by definition, with a high proliferation rate (Ki67 > 20%) combined with poor differentiation of their cells, resulting in an aggressive phenotype and poor prognosis [7]. PanNECs can occur with admixed components of other carcinoma types of non-neuroendocrine origin, this can be either ductal adenocarcinoma (PDAC) or acinar cell carcinoma (ACC). Mixed ACC NEC (MiNEN) are indeed a diagnostic challenge, reviewed by [8–10]. Within the high-grade subclass of PanNEN, 1/3 represents as a NET, while the remaining 2/3 are NECs (reviewed in [11]). Histologically, however, the distinction between G3 PanNETs and PanNECs remains difficult and ambiguous in a high number of cases, leading to misclassification [12–14].

All PanNENs are malignant tumors and pancreatic in origin; however, the field is rapidly acknowledging a clear distinction between poorly differentiated PanNECs and well-differentiated PanNETs [1]. Compared to PanNETs, PanNECs are highly aggressive and rapidly fatal, and most patients die within one year of diagnosis [15, 16]. PanNECs are also more responsive to platinum-based treatments than PanNETs, at least initially [17, 18]. Unlike PanNETs, which carry mutations in *MEN1*, *ATRX*, *DAXX*, PanNECs have a mutational profile similar to PDACs, characterized by genomic alterations in *KRAS*, *SMAD4*, and *TP53.* They additionally display loss of Rb1, further distinguishing them from PanNETs [19]. PDACs have been shown to originate from normal ductal or acinar cells [20, 21], while G1 and G2 PanNETs have been shown to originate from endocrine cells of the pancreas [22–24]. As yet, no study has attempted to identify the origin of PanNECs.

Several transcription factors are expressed and repressed in a spatio-temporal manner in order to form and maintain adult pancreatic cell types. For example, the transcription factor PDX1 is critical for maintaining β cells, while ARX, upstream of IRX2, is a well-established α-cell-specific transcription factor [25–31]. Presence of NKX6-1, NKX2-2, and PAX6 further maintains the endocrine lineage for α and β cells [32, 33]. SOX9, a downstream target of NOTCH, is required for the establishment of cell fates of endocrine and exocrine cells [34, 35]. While SOX9 is absent in committed endocrine precursors, it is an important player in pancreatic ductal and centro-acinar cell development but not in adult acinar cells [36, 37]. Importantly, Kopp et al. have shown a relationship between oncogenic *KRAS* and *SOX9* expression in the formation of premalignant PDAC lesions [38]. The sub-classification of PanNENs routinely relies on proliferation, morphological markers, immune phenotype, and symptoms associated with excessive hormone secretion. However, as most high-grade PanNETs and indeed all PanNECs are non-functional tumors, a more robust method for their distinction is required [39].

In this study we utilized genetic and epigenetic profiling, including Illumina 850K beadchip array methylation profile analysis, to classify PanNECs in relation to PanNETs of all grades and to identify the possible cell of origin of PanNECs. We find that PanNECs have DNA methylation profiles distinct from PanNETs, providing a means to distinguish histologically similar PanNECs and high-grade PanNETs. Furthermore, similarities between acinar cell and PanNEC methylation profiles point to an exocrine cell origin for this subtype of pancreatic carcinomas.

## Methods

### Patient cohort and experimental representation

**Fig. 1:**
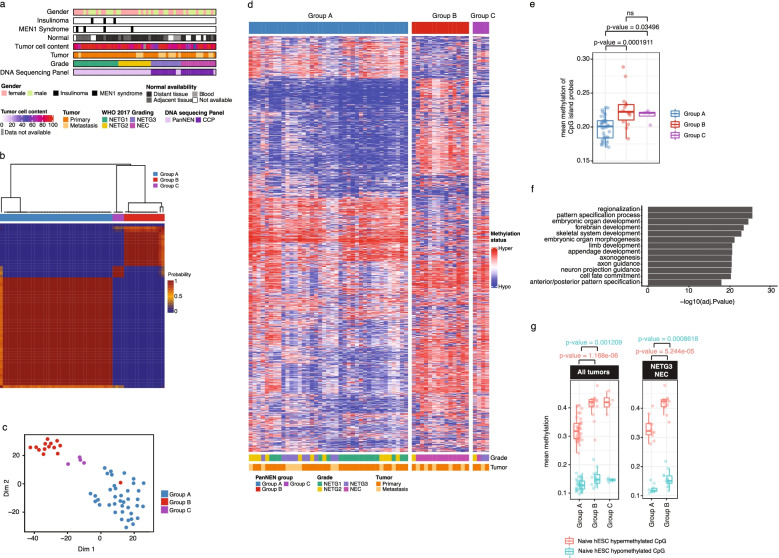
PanNENs subdivide into two main methylation groups. **a** Characterization of PanNEN cohort. WHO, World Health Organization; CCP, comprehensive cancer panel. **b** Unsupervised class discovery using 10,000 (10K) most variable methylation probes. Heatmap displays pairwise consensus values of the samples. **c** tSNE representation of PanNEN subgroups using 10K most variable probes. **d.** Heatmap displaying methylation status of 10K variable probes in each of the Groups A, B, and C. Methylation beta value was used to perform hierarchical clustering separately on each subgroup, identifying closely similar samples. Color range blue to red represents methylation beta value, columns indicate samples, and rows methylation probes. **e** Mean methylation of CpG island probes in PanNEN subgroups. Boxplot represents the distribution of mean methylation, each dot depicts a sample. **f** GO ontology analysis of 10K most variable probes, representing top 12 terms based on -Log10P-value. **g** Mean methylation of human Embryonic Stem Cells (hESC) associated hypermethylated and hypomethylated probes in PanNEN subgroups. Boxplot represents the distribution of hypermethylated (red) and hypomethylated (blue) CpG probes of hESC in cohort (left panel), or only in PanNETG3/ PanNEC samples from Group A and B respectively (right panel). Two-sample Wilcoxon test. Boxes show 25th and 75th percentiles and sample median as horizontal line, whiskers show maximum and minimum point

The cohort consists of 57 PanNEN samples collected from 55 patients (28 female and 27 male). Forty-one samples were collected from primary PanNENs and 16 from metastases. For one patient (PNET77), both primary and metastasis were obtained, and for another patient (PNET56), two metastases were obtained from two different time points, one year apart (Table 1). The Institute of Pathology at Charité - Universitätsmedizin Berlin provided 48 samples of all grades, and the University of Bern provided 9 NETG3/NEC samples (clinical information in Additional file 1: Table S1). All cases were classified according to the WHO 2019 criteria [6, 8]. Primary classification is based on morphology, with additional immunohistochemistry of Synaptophysin, Chromogranin A, P53 and RB1 for all cases, as well as Ki67 to analyze the proliferation index. All samples were collected as formalin-fixed paraffin-embedded (FFPE) blocks, and normal controls for the respective patients were collected, with the exception of 10 cases (Fig. 1a). Normal tissue sections were obtained as either tissue adjacent to the tumor (as per the pathologist’s examination) (normal adjacent *n* = 15, Additional file 2: Fig. S1b), or as a completely separate block containing only normal tissue (normal distant *n* = 29). Blood samples were available in 3 cases. All patients provided signed consent as part of the clinical documentation protocol of the Charité - Universitätsmedizin Berlin. Samples of the University of Bern were provided by the Tissue Biobank Bern (TBB) according to the relevant Ethics approvals.

**Table 1:** Cohort characteristics

Characteristics	Variable	Count	(%)
Gender	Female	27	47.4
	Male	20	35.1
	Unknown	10	17.5
Normal	Normal adjacent tissue	15	26.3
	Normal distant tissue	29	50.9
	No normal	10	17.5
	Peripheral blood	3	5.3
Tumor	Metastasis	16	28.1
	Primary	40	70.2
	Unknown	1	1.8
Location	Head	17	29.8
	Tail	14	24.6
	Liver	12	21.1
	Body	2	3.5
	Lymph node	2	3.5
	Multiple primary lesion location	2	3.5
	(Other)	8	14.0
Grade	NETG1	18	31.6
	NETG2	13	22.8
	NETG3	12	21.1
	NEC	14	24.6
Diagnosis	Insulinoma	3	5.3
	MEN1 Syndrome	4	7.0

### PanNEN panel design

We designed a custom panel targeting PanNEN relevant genes, covering all mutations associated with PanNENs. The design involved first performing a text-mining approach to extract high-quality information regarding genes associated with PanNENs from GeneView [40], the Catalogue of Somatic Mutations in Cancer (COSMIC) [41], and mutations collected from PanNEN publications [42–45]. From this list, 47 likely driver genes of PanNEN were selected, and amplicons were then designed for the GRCh37 genome by providing the Ion AmpliSeq Designer tool (Life Technologies) the candidate genes under the criteria “DNA Gene design (multi-pool)”. The panel was designed to generate primers targeting 125-bp stretches of exon regions of the selected genes. The complete panel included 1175 amplicons, divided into two pools (Additional file 2: Fig. S2a), a detailed list of genes and amplicons of the PanNEN NGS panel can be found in Additional file 1: Table S3. Sequencing results from the PanNEN panel in addition to a commercial panel (CCP, ThermoFisher) can be found in Additional file 1: Table S4.

### DNA isolation

Tissue samples were sectioned and stained with Hematoxylin and Eosin (H&E). Pathologists demarcated tumor and healthy tissue areas in the H&E slides, and depending on the size of the marked area, 12 sections of 5μm each from tumor samples and 6 sections of 5μm for control normal tissue were used for DNA isolation (Additional file 2: Fig. S1b). The tissue was macro-dissected from the slides and DNA was prepared using the GeneRead DNA FFPE kit (Qiagen, Netherlands). Quality and quantity of DNA was determined by RNAse P quantification (Thermo Fisher Scientific, USA).

### DNA sequencing

We used 20ng of DNA for library preparation using the Ion Ampliseq Library kit (Thermo Fisher Scientific). Regions were targeted by primers distributed into two amplicon pools per DNA sample for the PanNEN panel and 4 amplicon pools per DNA sample for Comprehensive Cancer Panel (CCP). Together, the panels covered 432 genes. Upon ligation to Ion Xpress Barcode Adapters (Thermo Fisher Scientific) and purification using Agencourt AMPure beads (Beckman Coulter), two samples were mixed at equal ratio on a 318v2 sequencing chip. Using the Ion Torrent PGM (Thermo Fisher Scientific), the samples were sequenced at an average read depth of 1158 reads using the PanNEN panel and an average read depth of 217.03 reads for CCP, in order to generate the raw intensity data.

### DNA methylation

DNA methylation profiling of all PanNEN cohort samples was performed with 200–500ng of DNA using the Infinium® MethylationEPIC BeadChip array (850K; Illumina, Inc., San Diego, CA, USA) according to the protocols provided by the manufacturer.

Normal cell type 450K methylation data of Pancreatic α, β, acinar and ductal cells were obtained directly from the published lab or GEO (Neiman et al. [46], GSE122126 [47], and GSE134217 [48]). In addition, 167 PDAC 450K methylation data was also obtained from GSE49149 [49]. We also obtained primed human embryonic stem cell (hESC) EPIC data from GSE128130. All datasets utilized are shown in Table 2.

**Table 2:** Source of external datasets used in our study

Sample ID	New sample annotation	Dataset
Alpha rep 1	Alpha A	Neiman et al.
Alpha rep 2	Alpha B	Neiman et al.
Beta rep 1	Beta A	GSE122126
Beta rep 2	Beta B	GSE122126
Beta rep 3	Beta C	Neiman et al.
Ductal 1	Ductal A	GSE134217
Ductal 2	Ductal B	GSE134217
Ductal rep 1	Ductal C	GSE122126
Acinar 1	Acinar A	GSE134217
Acinar 2	Acinar B	GSE134217
Acinar rep 1	Acinar C	GSE122126

### Immunohistochemistry (IHC)

Representative samples from Groups A and B were stained for ARX, PDX1, and SOX9. We used 2.5μm FFPE sections for ARX (1:1500, R&D Systems, sheep, AF7068), PDX1 (1:100, R&D Systems, mouse, MAB2419), and SOX9 (1:100, Cell Signaling, rabbit, mAb #82630) immunostainings. Antigen retrieval was performed (Tris30 buffer at 95°C for 30 min). The primary antibody was incubated for 30 minutes. Visualization was performed with a Bond Polymer Refine Detection kit, using DAB as chromogen (3,3′-Diaminobenzidine). Staining was assessed by pathologists and marked as strong (+++), moderate (++), weak (+) single cell positivity and is reported in Additional file 1: Table S1. The immunostaining for all antigens was performed on an automated staining system (Leica Bond RX; Leica Biosystems, Nunningen, Switzerland). All cases were examined by at least two pathologists (AJ, DH, AP). Reclassification was conducted after additional IHC had been performed (CD10, Trypsin, Cyto-Keratin, and β-Catenin).

### Data visualization and statistics

All data analysis, statistics and visualization was performed in R (version 4.0.0). Visualization was done using the base R plotting function, ggplot2 package or ComplexHeatmap package [50]. The appropriate statistics mentioned above were all performed using respective R packages or base R functions. For survival analysis, the “survival” and “survminer” packages were used [51, 52].

### Data processing and analysis

Detailed analysis is described in Additional file 2: supplementary methods.

## Results

### Sample characteristics

To investigate PanNENswe performed methylation analysis using the Infinium Methylation EPIC (850K) beadchip platform in addition to high-depth DNA panel sequencing of 57 PanNENs. The cohort consisted of 43 PanNETs including NETG1 (*n* = 18), NETG2 (*n* = 13), NETG3 (*n* = 12), and 14 PanNECs (Fig. 1a, representative H&E sections in Additional file 2: Fig. S1a, details in 1 and Additional file 1: Table S1).

### DNA methylation classification identifies a distinct PanNEC subgroup within PanNEN samples

To classify PanNEN tumors we performed unsupervised class discovery using the 10,000 (10K) most variable probes and defined three distinct groups at the methylation level: Groups A, B, and C (Fig. 1b; Additional file 2: Fig. S1c and methods for details). A t-distributed stochastic neighbor embedding (tSNE) analysis showed consistent segregation of the samples, affirming the presence of distinct methylation patterns in the groups identified (Fig. 1c). The methylation beta values from the 10K most variable probes revealed no distinction between the groups (Fig. 1d), as Group A was composed of 39 well-differentiated PanNETs of all grades including 10 NETG3, while Group B harbored 13 from a total of 14 PanNECs, and one NETG2 sample. Group C consisted of two NETG3s, one NETG2, and one NEC (Additional file 1: Table S1). Patient survival data confirms the distinct separation of Group A and B enriched in PanNET and PanNEC, respectively (Additional file 2: Fig. S1d). Mean CpG island methylation was significantly higher in Group B and Group C compared to Group A (Fig. 1e). Importantly, the methylation profiles separated high-grade NETG3 from NEC tumors, as they were assigned with high precision to Group A and Group B, respectively.

Gene Ontology (GO) analysis of the genes associated with the 10K probes significantly enriched for terms involved in biological processes associated with organ development (Additional file 1: Table S2; Fig. 1f). Recent work evaluating the transition of human embryonic stem cells (hESC) primed to a naive state demonstrated a gradual and s acquisition of CpG hypermethylation in genes associated with development, which was mirrored in multiple cancer entities [53]. We found that naive hESC-associated and hypermethylated CpGs were significantly more hypermethylated in Group B compared to Group A tumors (Fig. 1g left panel). A closer analysis of NETG3 samples in Group A compared to NEC samples from Group B maintained a similar significant difference in the distribution of hypermethylated CpGs of naive hESC (Fig. 1g right panel), highlighting the difference in developmental states between these histologically similar high-grade PanNEN subtypes.

### Distinct recurrent mutations separate PanNENs in Groups A and B

**Fig. 2:**
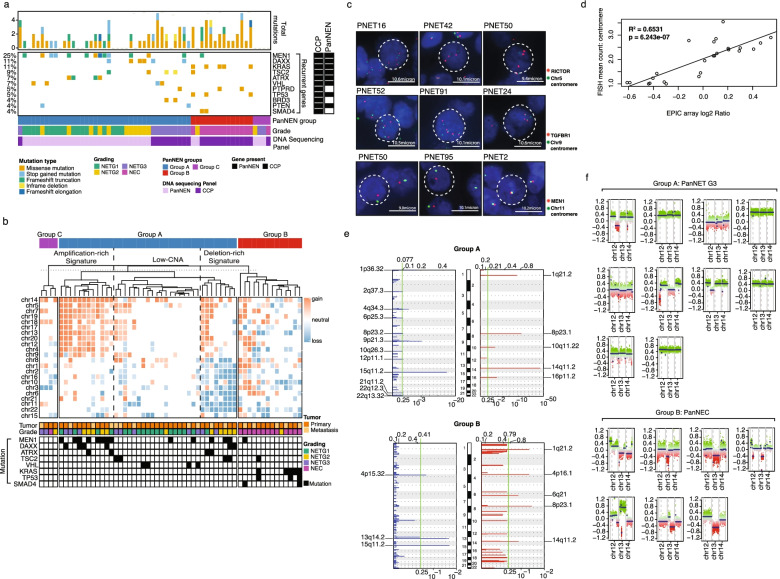
Genetic aberrations distinguish Group A and Group B. **a** Mutational landscape in PanNEN subgroups. Panel sequencing using in-house panel (PanNEN) and commercial cancer panel (CCP) Only genes mutated more than once are displayed here. Complete mutation profiles can be found in Additional file 2: Fig. S2b. Colors depict variant type (white spacing: no mutation identified). The DNA sequencing panel at the bottom depicts which targeted panel we used for the sample, while the ‘gene present’ annotation on the right side depicts whether the gene is present in the PanNEN panel or the CCP panel. Samples are sorted according to the PanNEN Groups; A, B and C. **b** Whole chromosomal aberrations in PanNEN subgroups. Hierarchical clustering of mean log2 ratios of chromosomal segments; dotted line represents cut-off used to identify amplification, low-CNA, and deletion-rich signatures; column annotation: tumor grade, tumor type and recurrently aberrated genes. **c**. Representative images of fluorescent in situ hybridization (FISH) validation. Red: gene probe. Green: centromere probe of chr5 (top panel), chr9 (middle panel), and chr11 (bottom panel). **d** Linear regression of mean copy number count of centromere derived from FISH (*y*-axis) and mean log2 ratios of chromosomal segments per autosome (*x*-axis); diagonal line: best fit model. *R*^2^ = 0.6531, *p*=6.232 × 10^−7^. **e** Focal aberrations in Group A (top panel) and Group B (bottom panel). Blue: focal copy number losses, red: focal copy number gain. Log2 ratio range at the top and *q*-value at the bottom of each graph. Green: *q*-value cut off at 0.25 to call significance. Significantly aberrated focal regions are identified. **f** Chromosome 12, 13, and 14 copy number status in NETG3 (top panel) and NEC (bottom panel). Intensity values of each bin are plotted in colored dots; each color indicates ‘methylated’ and ‘unmethylated’ channels of each CpG; segments are shown as horizontal blue lines

To further characterize the PanNEN samples at the mutational level, we employed high-depth panel sequencing utilizing the commercially available Comprehensive Cancer Panel (CCP), and a custom PanNEN panel (Additional file 2: Fig. S2a, Additional file 1: Table S3). Alterations characteristically associated with PanNETs were enriched in Group A, for example recurring mutations in *MEN1*, *DAXX*, *ATRX*, and *TSC2* in 13, 6, 4, and 4 of the 39 Group A samples, respectively. In contrast, mutations in *DAXX* and *ATRX* were absent in Group B, while only one Group B sample each was mutated in *MEN1* and *TSC2* (Fig. 2a shows recurring mutations; complete list in Additional file 2: Fig. S2b and Additional file 1: Table S4). In addition, four Group A samples contained aberrations in *VHL*, and two samples contained *PTEN* mutations. In contrast, *KRAS* (5) (with G12D, G12R, G12V, and Q61D mutations), *SMAD4* (2), and *TP53* (3) mutations were exclusively seen in Group B. In total, 16 samples, including the single non-PanNEC sample in Group B, did not harbor any known driver mutations detected by the DNA panels (all mutations discussed are either non-synonymous, deletions, or indels. Variant allele frequencies in Additional file 2: Fig. S2c). Two patients in our cohort had multiple samples: PNET77 and PNET56. Patient PNET77 had tissues of primary tumor and liver metastasis (PNET77P and PNET77M, both NETG3 samples in Group C) surgically removed two years apart. Neither sample displayed mutations covered by either of the panels. In contrast, Patient PNET56 had two liver metastasis samples in our cohort; PNET56P1 and P2 (both NETG3s in Group A) removed one year apart. Both samples carried the same alterations in *TSC2* and *BRD3*. Collectively, the mutational profiles uncovered key molecular distinctions between Group A and B samples, which are enriched for aberrations in *MEN1*, *DAXX*, and *ATRX* and *KRAS*, *TP53*, and *SMAD4*, respectively.

### Copy number alterations separate PanNECs from PanNETs

Within Group A we found three profiles of copy number alterations (CNAs): amplification-rich, deletion-rich, and low-CNA signature, displaying whole chromosomal copy number gains, losses, and mixed but limited aberrations respectively (Fig. 2b). Mean signal counts per sample from fluorescence in situ hybridization (FISH) and mean log2 ratio from CNA analysis were correlated and showed a regression coefficient of *R*^2^ =0.6531 and *p*=6.243 × 10^−7^ (Fig. 2c and Additional file 2: Fig. S3a and b). Recurrent mutations of tumor suppressor genes *MEN1*, *DAXX*, *TSC2*, and *VHL* were enriched in the amplification-rich and deletion-rich signature (Fig. 2b). The low-CNA signature contained few aberrations in most chromosomes with no clear recurrences, and this signature was a predominant feature of Group A NETG1 tumors. Tumors in Group B and Group C were defined by few recurrent whole chromosomal aberrations, likely due to the low number of samples in the groups.

We additionally investigated focal CNA using GISTIC (Fig. 2e). Chromosome regional gains of 1q21.2, 8p23.1, and 14q11.2, and deletion of chromosomal region 15q11.2 were significantly associated with both Group A and Group B. Group A had unique significant gains in chromosomal regions 10q11.22 and 16p11.2, which were not present in Group B. In contrast, only Group B showed focal gains in 4p16.1 and 6q21. Chromosomal deletions exclusive to Group A were in regions 1p36.32, 2q37.3, 4q34.3, 6p25.3, 8p23.2, 9p21.3, 10q26.3, 12p11.1, 21q11.2, 22q12.3, and 22q13.32, whereas Group B carried focal deletions in 4p15.32 and 13q14.2. Deletion of 9p21.3 in Group A resulted in loss of *CDKN1B and CDKN2A*, two key regulators of the cell cycle (Additional file 1: Table S5). Interestingly, deletion of 13q14.2 affecting *RB1*, also an important cell cycle regulator, was seen in 50% of Group B. (Fig. 2f, Additional file 1: Table S6). On closer examination, we found that NETG3s in Group A did not show losses of the *RB1* region (Fig. 2f lower panel). Therefore, while Group A is enriched for recurrent whole chromosomal and focal copy number aberrations, Group B exhibited significant focal aberrations and exclusively harbored *RB1* loss associated with PanNECs.

### PanNETs in Group A harbor endocrine cell of origin signatures

**Fig. 3:**
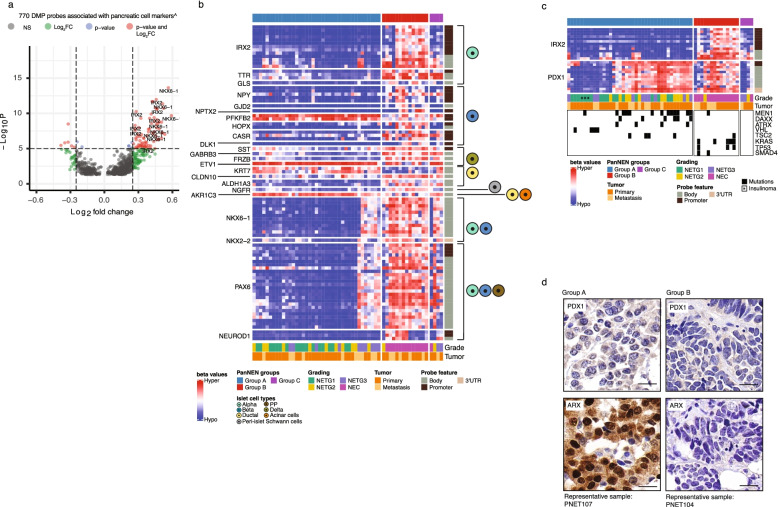
Cell marker analysis in PanNEN subgroups identifies endocrine features in Group A. **a** Differentially methylated probes (DMPs) associated with pancreatic cell markers (*n*=770 probes). Each point represents a DMP. Dotted line: intersect between -Log10 *P* value and the log2 fold change (FC) for a given probe. Cut-off for significance: -Log10 *P*-value > 5 (adjusted *p*-value: 10^−6^) and log2FC: >|0.25|. Red: probes passing both cut-offs; green: probes only passing the log2FC threshold; blue: probes with only have a significant *p*-value; gray: probes that did not pass any of the cut-offs; Significantly associated DMP probes of IRX2 and NKX6-1 are labeled. **b** Methylation beta value of significant DMPs of pancreatic cell markers. Heatmap displaying the methylation beta values of DMPs (row) in each sample (column). Pancreatic cell-types which the cell marker is associated with, according to PanglaoDB, are displayed on the right. **c** Methylation beta value of probes associated with *IRX2* and *PDX1*. DMP probes of *IRX2* and 10K probes associated with *PDX1* (rows) for each sample. Lower panel depicts recurrently mutated genes. **d** Representative IHC of *ARX* and *PDX1* in PanNEN subgroups. Scale bar: 20μm

Cell-of-origin studies in PanNEN have been thus far restricted to well-differentiated PanNETs. Therefore, we investigated cell-of-origin methylation patterns in our diverse PanNEN cohort. First, we curated a list of 174 markers from PanglaoDB [33] uniquely expressed in each differentiated cell type of the adult pancreas (Additional file 1: Table S7). We called differentially methylated probes (DMPs) from our samples (Additional file 1: Table S8) and identified genes overlapping with the curated list. We identified 85 significantly enriched probes (red points in Fig. 3a, Additional file 2: Fig. S4a, Additional file 1: Table S9) associated with 23 markers of α, β, ɣ, δ, ductal, acinar and Islet Schwann cells (Additional file 2: Fig. S3d). Among these, α cell markers such as *IRX2*, *TTR*, and *GLS* were hypomethylated across Group A samples (Fig. 3b). IRX2 and PDX1 characterize α-like and β-like tumors, respectively [22, 25–27]. Group A tumors were consistently hypomethylated in promoter-associated probes of *IRX2*, while Group B showed strong hypermethylation (Fig. 3a, b). Although *PDX1* was not differentially methylated between Group A and Group B, the 10K probes establishing the groups displayed variable methylation patterns of PDX1 within the groups (Fig. 3c and Additional file 2: Fig. S3e for sample IDs). Group A tumors separated into two subgroups with respect to *IRX2* and *PDX1* methylation status, whereby one subgroup carried hypomethylation of both *IRX2* and *PDX1*, while in the second subgroup *PDX1* was hypermethylated. The latter subgroup was enriched for *MEN1*, *DAXX*, and *ATRX* mutations, while recurring *VHL* mutations were seen in the prior subgroup (Fig. 3c). The transcription factor ARX was also found in a subgroup of Group A tumors and ARX+PDX1− phenotype was significantly more common when compared to Group B (*p*-value=0.02036: Fisher’s exact test) (Fig. 3d and Additional file 2: Fig. S4). In addition, genes associated with endocrine cell lineage maintenance, such as *PAX6*, *NKX6-1*, and *NKX2-2* were mainly hypomethylated in Group A, except for NETG3 samples (PNET42, PNET57, PNET61, PNET56P1, PNET56P2, and PNET107), and one NETG2 (PNET24), which showed hypermethylation of *PAX6*, *NKX6-1*, and *NKX2-2* genes similar to Group B samples (Fig. 3b). In contrast, Group B, comprising almost exclusively PanNECs, was characterized by hypermethylation of all DMPs under investigation, with the exception of *KRT7*.

### PanNECs in Group B display an acinar-like cell signature and differ from PDACs in ductal-like cell signatures

**Fig. 4:**
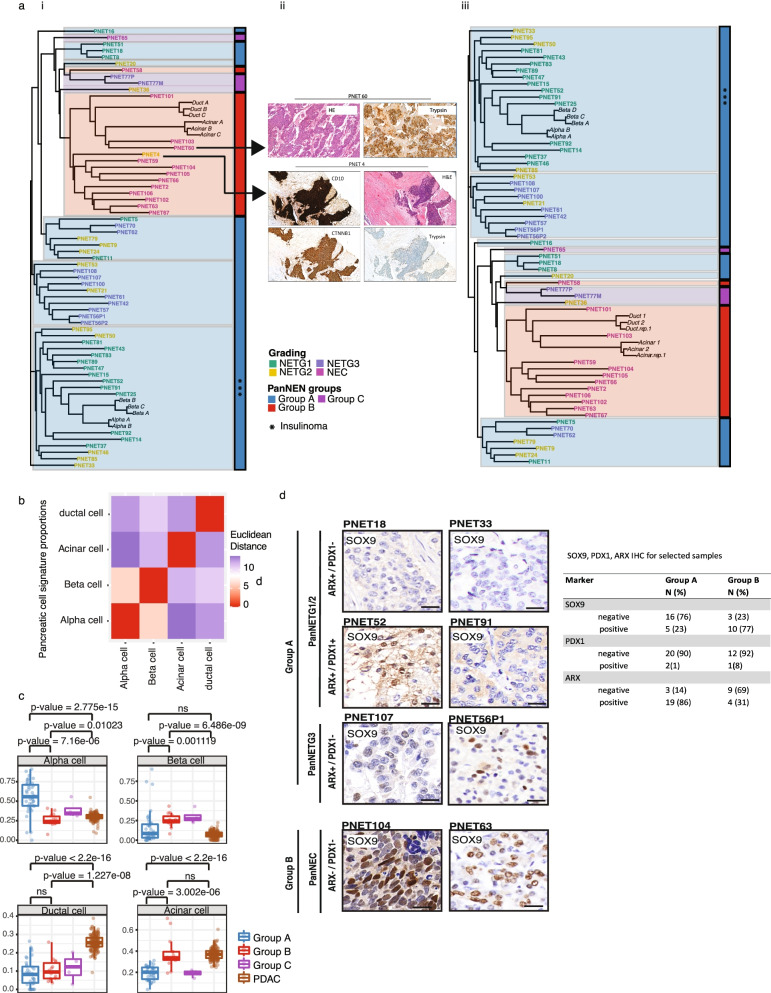
Cell-of-origin analysis using normal cell type methylation profiles and SOX9 in PanNEN subgroups indicate exocrine lineage for Group B tumors. **a** (i) Phylo-epigenetic analysis of PanNEN tumors and normal pancreatic cell types. Pearson distance between the samples computed using differentially methylated CpGs between normal α-, β-, ductal and acinar cells (*n* = 46,500, adj. *p* value < 0.01 and |Δβ | > 0.2) and neighbor-joining tree estimation. (ii) IHC of outliers PNET4 and PNET 60, which were re-assessed and removed from phylo-epigenetic analysis (i). (iii) New phylo-epigenetic analysis without PNET4 and PNET60. **b** Euclidean distance between each cell type was computed and correlation matrix of the distances is displayed. Heatmap depicts the distance between a given normal cell-type pair. **c** Boxplot representing distribution of the proportion of atlas signatures of α-, β-, ductal and acinar cells (each main box) in the subgroups and PDACs; each dot depicts the proportion of atlas signatures of the respective cell type in a given sample. Two-sample Wilcoxon test. **d** IHC of SOX9. Representative images for each subgroup; scale bar: 20μm. Table depicts the total IHC score for Group A and Group B samples

In order to identify the cell of origin for samples from Group B, we extended our analyses to include normal pancreatic methylation profiles. We obtained Illumina 450K array methylation profiles of presorted normal pancreatic cell types: α (*n* =2), β (*n* =3), acinar (*n* =3), and ductal (*n* =3) cells [46, 48]. We identified 46,500 DMPs differentiating the cell types from one another (adjusted *P* value < 0.01, absolute Δ beta > 0.2, Additional file 1: Table S10 to S15). We determined Pearson distance between the tumor samples and the pancreatic cell types using these DMPs and constructed a phylo-epigenetic tree (Fig. 4a). Two main branches separate the whole cohort; the lower branch consists completely of Group A and clustered closely with β and α cells. The insulinomas grouped together and maintained the closest distance to the normal endocrine cell type. Strikingly, all tumors from Group B, except PNET58 an ARX+ and PDX1+ (Additional file 2: Fig. S4d), distinctly clustered with ductal and acinar cells, forming a separate clade together, indicating a clear resemblance to the exocrine cells of the pancreas and evidently distant from the endocrine cells of the pancreas.

We next applied an independent method to determine the normal cell signature composition of our PanNEN samples. As a reference, we utilized 6096 CpG probes which distinguish ductal, acinar, and β cells [47]. For the missing α cell reference we utilized the 450K profiles mentioned in the previous section [46] and determined the methylation values for the CpGs which differentiated β, acinar, and ductal cells (Additional file 1: Table S16). Using Euclidean distance analysis, we confirmed that the reference cell types were clearly discerned from one another (Fig. 4b). Given the reference and the tumor samples, we ran a deconvolution algorithm which models the profile of the tumors as a linear combination of the methylation profiles of the cell types (Additional file 1: supplementary methods). This method determines the methylation signature proportion using non-negative least squares linear regression (NNLS). We additionally added 167 PDACs as an independent pancreatic tumor type [54]. Group A PanNETs displayed significant enrichment of α-cell signature composition compared to Group B, and to a lesser extent to Group C (Fig. 4c, Additional file 2: Fig. S5a, b, Additional file 1: Table S17 and S18). Group B NECs, on the other hand, showed a significant increase in acinar cell signature proportion when compared to Group A and to Group C. Interestingly, the acinar cell composition of Group B was similar to that found in PDAC tumors. However, the ductal signature composition of PDAC tumors was significantly higher, distinguishing this pancreatic exocrine cancer from PanNENs and specifically from Group B. NETG3 samples from Group A contained similar profiles to other PanNET samples, and showed no resemblance to the acinar cell similarity of PanNECs (Additional file 2: Fig. S5c). Using the same method, we further looked at the two subgroups within Group A, which showed differences in *PDX1* methylation patterns (Fig. 3c). We found an α-like cell profile signature more significantly enriched in samples with hypermethylation of *PDX1*, and a β-like, intermediate cell profile in samples with hypomethylation of both *PDX1* and *ARX* (Additional file 2: Fig. S5d).

To determine the key probes that establish the resemblance of Group B to the exocrine cells and distinguishing them from the endocrine cells as well as Group A and C samples, we determined the 10K most variable probes from the aforementioned DMPs and performed an unsupervised clustering using ConcensusClusterPlus [55]. We computed the mean methylation of each probe in the two clusters from the new analysis; cluster 1 and cluster 2 (Additional file 2: Fig. S6). Next, we determined the distribution of the absolute difference in the mean methylation between cluster 1 and cluster 2 (ranging between 0.0001 to 0.5). To determine the highest varying probes between the clusters, and thereby the genes that establish the resemblance within cluster 2, we placed a cut-off for the absolute difference in mean methylation at 0.4 and higher (Additional file 2: Fig. S6a-d). We further focused only on the probes which were associated with the promoters (TSS200, TSS1500, 5′UTR, and 1st Exonic) to identify potential genes of interest. Genes that were hypomethylated or hypermethylated in the promoter regions within Group B samples and exocrine cells can be found in Additional file 1: Table S19.

The phylo-epigenetic analysis (Fig. 4a) as well as the cell signature composition analysis (Fig S5a) led us to reassess several outlier samples: PNET4 was the only NET sample found within Group B, PNET60 and PNET103 were seen to consist of a high proportion of acinar cells (Additional file 2: Fig. S5a), as well as all Group C samples, whose relationship seemed unclear. Therefore, we stained all Group B samples with Trypsin, a marker for acinar cell carcinoma of the pancreas (ACC), and all Group C samples with Keratin to exclude paraganglioma. PNET4 was stained with CD10 to control for pseudopapillary neoplasia (SPN), ß-catenin, and cytokeratin to exclude paraganglioma.

This re-analysis revealed that PNET60 is most likely an ACC, while 3 other Group B samples (PNETs 101, 103, 106) are tumors of mixed identity (MiNEN), consisting of different proportions of ACC and NETs (Additional file 1: Table S1). PNET4 was identified as a likely SPN being strongly positive for CD10 and ß-catenin (Fig. 4a (i, ii)). Based on these results, we excluded PNET60 and PNET4 from our cohort and re-conducted the phylo-epigenetic analysis, which showed the identical tree phylogeny (Fig. 4a (iii)).

### Group B PanNECs display SOX9 patterns similar to exocrine cells

The pattern of Group B acinar similarity to PDACs compelled us to investigate whether this similarity was SOX9 dependent [38]. We performed IHC of SOX9 for representative samples of the cohort. From 11 Group B PanNECs 9 samples were positive for SOX9 staining (Fig. 4d, Additional file 2: Fig. S4e). In Group A, 4 from 17 samples (three NETG3s and one NETG2 (an insulinoma and a *MEN1* syndrome tumor)) displayed staining for SOX9, albeit with a low and heterogenous pattern, unlike Group B positive tumors (Fig. 4d and Additional file 2: Fig. S4f). We analyzed IHC of ARX and PDX1, with regard to *SOX9* expression, on the same samples. The Group A SOX9+ NETG3 were additionally ARX+. Three Group B SOX9+ samples were additionally ARX+. The SOX9- Group B sample was however ARX- and PDX1- (Additional file 2: Fig. S4f). Our IHC analyses showed that α-/β- like tumors in Group A harbored significantly more ARX+PDX1-SOX9, while Group B acinar-like tumors were enriched for ARX-PDX1-SOX9+ features (adjusted *p*-value = 0.001998: Fisher’s exact test and fdr corrected).

## Discussion

We report a methylation-based classification which accurately distinguishes PanNECs from all PanNETs, including G3 PanNETs. Our study demonstrates a potential exocrine cell of origin for PanNECs, distinct from the endocrine cell of origin of PanNETs. Our findings lead to a novel approach, extending the classical histopathological diagnosis by an epigenomic diagnosis of two otherwise histologically challenging aggressive subsets of PanNEN. Moreover, we find tumor cell methylation profiles can indicate misdiagnosed tumors and potential cells of origin.

DNA methylation at CpG dinucleotides is a mechanism of cell-type-specific gene regulation inherited in a continuous manner throughout development, hence it is a robust marker of cell identity [56]. Methylation patterns are now considered as a robust method to identify tumor cell-of-origins across tissues [57], and in different cancer types [48, 58–61]. In addition, they have been used for characterization of subgroups within a tumor entity [62, 63]. Furthermore, methylation analysis has shown the significance of hypermethylation during the transition of committed cells to a naive stem cell state [53]. The cell type from which a cancer originates is highly informative to its identification, classification, treatment, and prognosis [64].

Our investigation of 10K most variable probes clustered a PanNEN cohort, representing all tumor grades, into three groups. The largest two groups, Groups A and B, clearly distinguished NECs from the rest of the cohort. Three recent studies have investigated PanNET subgroups at the epigenetic level [22–24]. Cejas et al. investigated histone acetylation and transcriptomes of pancreatic NET samples [22]. Di Domenico et al. [23] and Lakis et al. [24] used 450K methylation profiles from cohorts of well-differentiated PanNET samples. Our work expands the field by including the most aggressive subtypes of PanNENs: NETG3 and NEC tumors, which the aforementioned studies largely excluded. In addition, we used the 850K EPIC beadchip assay, significantly increasing the number of CpGs investigated. All three studies assigned the cell of origin of early PanNETs to α and β cells of the pancreas. These findings are reflected in our analysis of tumors belonging to Group A. Due to the inclusion of more high-grade NETG3 and poorly differentiated, high-grade PanNEC samples in our study, the methylation patterns separate the groups into more encompassing subgroups, placing those of low grade, and α and β similarity together, distinct from the undifferentiated PanNECs (Fig. 1d).

Phylo-epigenetic analysis exposed the tight clustering of PanNEC samples with exocrine cells, and their separation from endocrine cells and their relationship to PanNETs (Fig. 4a). PanNECs have been repeatedly compared to PDACs in terms of mutational spectrum [19]. The key difference becomes obvious at the cell-type similarity level, whereby PDACs were clearly similar to ductal and acinar cells, whereas NEC profiles were only similar to acinar cell profiles (Fig. 4c). A recent study by Kopp et al. showed that SOX9 accelerated the formation of precursor lesions of PDAC when co-expressed with oncogenic KRAS. By lineage tracing, their study also suggested that upon the expression of *SOX9*, pancreatic intraepithelial neoplasia (PanIN) lesions and subsequently pancreatic ductal adenocarcinoma arise from ductal metaplasia of the pancreatic acinar cells, a phenomenon known as acinar-to-ductal metaplasia [38]. SOX9 is a crucial factor regulating pancreatic cell development, initially maintained in the multipotent progenitor state [26, 37], and subsequently restricted to NKX6.1+ bipotent progenitor cells. Later, in adult pancreatic cell types, SOX9 is constrained to ductal and centro-acinar cells of the pancreas [65, 66]. We detected SOX9 in 81% (9/11) of PanNECs available for IHC in Group B and 60% (3/5) of NETG3 samples in Group A. Interestingly, Group B samples carried a SOX9+, ARX−, and PDX1− phenotype (Additional file 2: Fig. S4b) and mirrored the naive hESC methylation profile (Fig. 1g). In line with the aforementioned findings in PDAC, our data led us to hypothesize that Group B samples are acinar-like tumors which undergo a mechanism similar to that of PDAC formation via the expression of *SOX9*. In contrast, NETG3 SOX9+ samples found in Group A carried a profile similar to α cells, but not to acinar cells (Additional file 2: Fig. S5c). Since SOX9 plays a critical role in the multipotent and bipotent state of pancreatic development, these NETG3 tumors may originate from endocrine cells, given their similarity to α and β cells, and in the course of tumor progression revert to expressing SOX9 as a mechanism to move towards a progenitor-like phenotype.

PanNEC mutational patterns such as *KRAS*, *SMAD4*, and *TP53* were present in Group B. Using focal DNA copy number analysis we found that RB1 loss, shown in NECs at the protein level [67], was due to DNA copy number loss of chromosome 13, found in 50% of the PanNECs but not in any NETG3 samples (Fig. 2f). PanNEC subgroups with mutant KRAS and RB1 loss showed a higher response rate to first line platinum-based treatment, but with a shorter overall survival rate [68]. In contrast, cell cycle regulators *CDKN1B* and *CDKN2A* were lost in Group A PanNET samples.

Group A tumors were all PanNETs and included all grades G1, G2, and G3 with mutated *ATRX*, *DAXX ,*and *MEN1* (A-D-M) genotypes as well as known copy number patterns for PanNET tumors [69]. The top DMPs in pancreatic cell markers belonged to *IRX2* and *NKX6-1*, and maintained a strict hypomethylation in Group A, strongly indicative of an endocrine origin (Fig. 3b), and a closer look at their methylation pattern in Group A clearly separated the samples into an α-like subgroup (*PDX1* hypermethylation and *IRX2* hypomethylation), and a β-like and intermediate subgroup of endocrine-like tumors (*PDX1* hypomethylation and *IRX2* hypomethylation) that carry >75% β cell signature or an equal proportion of α, β cell signature, respectively (Fig. 3c; Additional file 2: Fig. S5d). The mutational characteristics of α-like tumors identified by Chan et al. is reflected in our results, where the majority of samples with A-D-M mutations show promoter hypermethylation in *PDX1* and hypomethylation in the *IRX2* gene probes and strong alpha-cell type signature [70] (Fig. 3c; Additional file 2: Fig. S5d).

Group C tumors carried no driver mutations and displayed a methylation pattern of CpGs associated with cell markers resembling a mixed Group A and B profile (Fig. 3b). Although they remain close to Group B samples in the phylo-epigenetic analysis (Fig. 4a), their similarity to acinar cell signatures is not comparable to that found in Group B (Fig. 4c). Despite the small size of Group C, further Keratin IHC confirmed their PanNEN diagnosis (Additional file 1: Table S1), yet we cannot define at the moment whether this small group is a true biological subgroup.

Strikingly, despite the limited number of samples, our methylation profiling was able to correctly exclude sample PNET4, a tumor initially diagnosed as PanNET, from other PanNET samples in Group A, where it was the single, not NEC sample in Group B. We re-assessed the identity of this sample using multiple markers (Fig. 4a (ii)), and found out, retrospectively, that it was indeed initially misdiagnosed as a PanNET, and is actually a solid pseudopapillary neoplasm (SPN). Our study therefore not only shows that methylation profiling distinguishes PanNETG3 from PanNEC, but also provides useful indications towards potentially misdiagnosed PanNENs.

## Conclusions

Our work establishes an exocrine cell of origin for PanNECs, resembling an acinar cell type. Methylation profiling is a superior method of tumor-type identification to genomic mutations, copy number alterations, or IHC of single markers. This is due to the epigenetic memory of the cancer’s cell of origin, as discovered in several studies [71, 72], most importantly in Cancers of Unknown Primary (CUP) [47, 61]. Epigenetic identification of cancer cell-of-origin can determine the diagnosis [23, 62], evolution [73–75], and treatment [68, 76] of this disease. Our study supports the introduction of methylation analysis to routine diagnosis of high-grade PanNEN tumors.

## Supplementary Information

**Additional file 1.** Supplementary tables S1-S19.

**Additional file 2.** Supplementary methods, and supplementary figures Fig S1-S6.

## Data Availability

Datasets (raw data files for DNA panel sequencing and EPIC methylation array) from this study are available in the European-Genome Phenome Archive under study number EGAS00001005731 at https://ega-archive.org/studies/EGAS00001005731 [77].
