# Supplementary material for: DNA methylation reveals distinct cells of origin for pancreatic neuroendocrine carcinomas and pancreatic neuroendocrine tumors
Source: Genome Med. 2022 Mar 1;14:24. doi: 10.1186/s13073-022-01018-w (PMC8886788; doi:10.1186/s13073-022-01018-w)
Supplement: Supplementary file 2 — Additional file 2. Supplementary methods, and supplementary figures Fig S1-S6. [file 13073_2022_1018_MOESM2_ESM.pdf]

Supplementary Methods

Data processing and analysis

Mutational analysis

The raw reads were first aligned to GRCh37 sequence using the Torrent Mapping and Alignment Program (TMAP; Life Technologies), placing a cut-off of > 50 nucleotides in aligned reads and a mapping quality of > 4 using an in-house python script. The processed bam files were then utilized for variant calling using TS Variant Caller plugin under the “strict” setting per Ion Suite (Ion Torrent platform) parameter profiles. The generated variant call format (VCF) files of the tumor-normal pair per patient was merged and reference and alteration read count for all variants within the two VCF files were extracted to determine the representation of the variants in both cases. The merged VCF file was then annotated using the SoFIA[1] annotation framework. Downstream filtering included removing variants that were positive for the following set of parameters: intronic and synonymous variants, 1000Genome variants with frequency greater than 1% in the population, variants within homopolymer regions > 4 nucleotides, tumor variant allelic frequency < 5% and finally, variants that were represented at comparable allelic frequencies in the matched normal tissue.

DNA methylation preprocessing

Raw idat files were preprocessed using subset-quantile within array normalization (SWAN) provided through the R package minfi [2,3]. Probes performing poorly in the analysis were further filtered out. The failed probes were identified if their detection p value was > 0.01 in at least one sample. Probes cross reactive to multiple sites in the genome[4], Sex chromosome probes and probes containing SNPs with an allele frequency > 0.01 were also filtered out.

When comparing 450K and 850K samples, the processing was done by first converting the 850K platform to 450K and then performing the aforementioned steps. When tumor and normal data were analyzed together, normalization was done using the preprocessFunnorm() function from the minfi package in order to accommodate for the global variation between normal and tumor data. This also entailed removing cross reactive probes of EPIC and 450K array (https://github.com/sirselim/illumina450k_filtering). hESC data normalization was performed as depicted in Patani, *et al,* 2020. Briefly, we normalized the data using the preprocessNoob() function from minfi and performed the aforementioned filtering approach to remove problematic probes. Finally, beta values for each dataset were used for all downstream analysis, statistics and visualization.

Subgroup identification and associated analysis

Using the 10K most variable probes identified by determining row (probe) standard deviation (σ), DNA methylation-based classes of PanNEN were identified with the R package ConsensusClusterPlus under the following parameters: maxK=12, reps=1000, pItem=0.8 and pFeature=1. The function performed agglomerative hierarchical clustering after performing 1-Pearson correlation distance. A consensus matrix carrying pairwise consensus values was finally generated for 12 clusters. The most stable number of clusters was determined based on the cumulative distribution score curve (CDF) that reached an approximate maximum (k=3) and the correlation heatmap for each 3-mer. Hierarchical clustering of 10K variable probes was done by first obtaining a dissimilarity matrix using an Euclidean algorithm and then performing the clustering using complete linkage. Hierarchical clustering was done using the R package “Stats” and tSNE was done using the “Rtsne” package under the perplexity=8. Genes associated with the 10K most variable probes were evaluated for GO pathway [57] biological processes term enrichment using the enrichGO() function in the clusterprofiler R package. The analysis was done under the following parameters: pAdjustMethod = “BH” (Benjamini and Hochberg), pvalueCutoff = 0.01, qvalueCutoff = 0.05. All genes represented in the Illumina EPIC array were used as

background. In order to reduce generality of GO terms, the simplify() function was used. The final set of terms was curated by filtering only those that showed an adjusted p-value less than 0.05 and fold enrichment of greater than 1.5. -$Log_{10}P$ value was calculated for the remaining terms and a barplot was generated for the 12 most significant terms using the ggplot2 package.

Differentially methylated probes (DMP) and associated analysis

Upon extracting and assigning the samples to the identified stable clusters Group A and Group B, differentially methylated probes (DMP) were identified out of all the CpG sites (upon the aforementioned preprocessing) using CHAMP package[5] function champ.DMP() under the following parameters: adjPVal = 0.05, and adjust.method = "BH", arraytype="EPIC".

Differentiated pancreatic cell markers were curated from PangaloDB (https://panglaodb.se/). Cell markers showing sensitivity_human > 0.05 for α, β, ɣ, δ, Epsilon, Acinar, Ductal and Islet Schwann cells were obtained. DMP associated genes that overlapped with curated Islet cell markers were extracted. In total, we detected 122 markers associated with the 770 DMPs from our samples. Final lists of DMP associated Islet cell markers were identified if they met the following criteria: abs(Δbeta) > 0.25 and -$log_{10}P > 5$. To determine closely related samples within each group, hierarchical clustering with complete linkage was performed using beta values of the identified Islet cell markers associated with DMPs before visualization.

DMP between α, β, ductal and acinar cell types were identified using CHAMP package function champ.DMP under the following parameters: adjPVal = 0.05, and adjust.method = "BH", arraytype="450K". Significant probes of each DMP set showing absolute Δbeta value > 0.2 and adjusted p-value < 0.01 were obtained. A total of 46,500 unique probe IDs were collected and defined as DMPs of normal cell types. Upon preprocessing and downstream filtering (see DNA methylation preprocessing section) of tumor and normal data combined, 38892 DMPs of normal cell type overlapping in tumor-normal matrix remained and methylation values were extracted to calculate Pearson distance using the function get_dist() from factoExtra R package. Finally,

neighbor-joining tree estimation was performed using nj() function in the ape package to generate phylo-epigenetic trees.

hESC probe identification was performed using an adaptation of method in Patani, *et al.,* 2020. Briefly, hESC probes carrying a mean beta < 0.3 across the primed cells were retained as background matrix. Unmethylated probes of hESC were then defined as those that carry mean beta < 0.3 in both primed and naïve hESC. Hypermethylated probes of hESC were defined by first calling DMPs using the background matrix. CHAMP.DMP() ran with the adjPval=0.05, adjust.method="BH", and arraytype="EPIC". Probes carrying Δbeta < -0.1 (hypermethylation in naïve state compared to primed state) were then extracted. Finally, to compare to the tumor samples, after normalization, preprocessing and filtering (see above section: DNA methylation preprocessing), the methylation values for unmethylated probes of hESC and hypermethylated probes of hESC in the tumors were determined and the mean per probe type in each sample was computed. The distribution of these computed mean per Group was visualized. In addition, mean values for NETG3 and NEC samples of Group A and Group B were extracted and separately visualized.

Normal cell signature analysis

In order to determine cell signature proportion in each sample, the methodology provided by Moss et al. was utilized [6]. Briefly, first the reference atlas (https://github.com/nloyfer/meth_atlas) was obtained, then just the β, ductal and acinar cell profiles and their featured CpGs were extracted. In order to add an α-cell profile, the normal cell types were preprocessed and normalized (Neiman et al., GSE122126 and GSE134217), and the α cells were extracted. The mean value of each probe was calculated and the overlap of the probes compared to the featured CpGs of Moss et al. were obtained. The final matrix of the normal reference "atlas" contained the methylation values for α, β, ductal and acinar cells of the overlapping probes. The euclidean distance between each sample given the probes was computed using get_dist() function from the FactoExtra R package. PanNEN and PDAC data were normalized separately as mentioned above, and the

methylation beta value matrix was transformed for subsequent analysis. The program developed by Moss et al. (https://github.com/nloyfer/meth_atlas/blob/master/deconvolve.py) was then employed to identify the normal cell signature proportion in samples of the PanNEN and PDAC cohorts (Additional file 1: Table S17 and S19).

Copy Number Aberrations (CNA)

CNA was identified from EPIC array data using the R package conumee (https://bioconductor.org/packages/release/bioc/html/conumee.html). Upon raw preprocessing, Mean log2 ratios of similar CNA segments per autosomal region were inferred from methylation signal intensities and a mean value per autosome was calculated for each sample to determine the log2 ratio of intensities across the chromosome. For analysis of whole chromosomal copy number changes within each group, we calculated the average log2 ratio of CNA segments per chromosome. A cut-off of $x > 0.15$ and $x < -0.15$ was placed to limit the number of false positives obtained upon comparing log2 ratio values to FISH count (Fig. 2d). To determine the subgroups within the cohort, euclidean hierarchical clustering was also performed on the data. To determine focal aberrations, the chromosomal segment log2 values, determined by conumee were obtained for samples of each group and were separately run in GISTIC software under the following parameters: -genegistic 1, -smallmem 1, -broad 1, -brlen 0.5, -conf 0.90 -armpeel 1 and -gcm extreme. GISTIC was only performed in Group A and Group B, and not for Group C, due to the limited number of samples.

Sanger Sequencing

To validate results from targeted massive parallel sequencing we performed Sanger sequencing on specific mutations. The resulting signal intensity images were manually scanned to identify the targeted mutations (Additional file 1: Table S4).

Fluorescence in-situ hybridization (FISH)

Fluorescence in situ hybridization (FISH) was performed on 3µm tumor sections from 23 samples. For detecting chromosome 5 we used a *RICTOR* gene probe, chromosome 9 with *TGFBR1* gene, and chromosome 11 with *MEN1* gene (Empire Genomics, USA). Hybridization was performed according to manufacturer's instructions. Where possible, we scored 40 cells per sample using an Olympus microscope. Analysis was conducted using 'BioView solo' (Abbott Molecular).

Bibliography

1. Childs, L. H., Mamlouk, S., Brandt, J., Sers, C. & Leser, U. SoFIA: a data integration framework for annotating high-throughput datasets. *Bioinformatics* **32**, 2590–2597 (2016).
2. Aryee, M. J. *et al.* Minfi: a flexible and comprehensive Bioconductor package for the analysis of Infinium DNA methylation microarrays. *Bioinformatics* **30**, 1363 (2014).
3. Maksimovic, J., Gordon, L. & Oshlack, A. SWAN: Subset-quantile Within Array Normalization for Illumina Infinium HumanMethylation450 BeadChips. *Genome Biol.* **13**, 1–12 (2012).
4. Chen, Y.-A. *et al.* Discovery of cross-reactive probes and polymorphic CpGs in the Illumina Infinium HumanMethylation450 microarray. *Epigenetics* **8**, 203–209 (2013).
5. Tian, Y. *et al.* ChAMP: updated methylation analysis pipeline for Illumina BeadChips. *Bioinformatics* **33**, 3982–3984 (2017).
6. Moss, J. *et al.* Comprehensive human cell-type methylation atlas reveals origins of circulating cell-free DNA in health and disease. *Nat. Commun.* **9**, 5068 (2018).

Figure legends

Supplementary Figure legends

Fig. S1| Three main subgroups define the PanNEN cohort. a. Representative H&E stainings for each grade. Scale bar: 50μm. **b.** Representative macro images of tissue H&E sections used for downstream analysis. Solid and dotted lines represent tumor and normal regions respectively, by pathologist's evaluation. **d.** Cumulative distribution function (CDF) curve of the resulting 'k-mer' count for 12-k's. Cluster count of 'three' resulted in the most stable number subgroups. **c**. Kaplan Meier survival graph from available patient data. Left: graph shows cohort according to tumor grade (P = 0.062). Right: graph shows cohort according to methylation groups A, B and C. Significant difference found between Group A and B (p = 0.0049) and between all groups (p = 0.011). P value generated using log-rank sum test statistics by survminer R package.

Fig. S2| Distinct mutational aberrations define PanNEN subgroups. a. Custom PanNEN gene panel for mutation analysis. Targeted genes of PanNEN panel are displayed in their respective chromosomal region. Insert: Overlap of PanNEN panel and commercial Comprehensive Cancer Panel (CCP). **b**. Mutational profile of PanNEN cohort ordered according to the most frequently altered gene. Top panel: barplot depicting mutational frequency in a given sample, colored according to variant type. Left panel: percentage value per row, frequency at which the respective gene is aberrated in the cohort. Right annotation: two row annotations displaying whether the respective gene was covered by the PanNEN panel, CCP panel or both. **c**. Allelic frequency of the mutations in the PanNEN cohort. Mutated genes are displayed at the bottom; Heatmap color represents the frequency at which the alterations were identified in a given sample (range from: 0.05 [blue], to 0.93 [red]). Grey: absence of aberration in the respective gene for a given sample. **d**. Frequency of most recurrently altered genes in Group A and Group B. The bars represent the

count of alterations of recurrently mutated genes (cut-off: 4% or 2 alterations). Fisher exact test was performed the altered gene was present in both Group A and Group B. P-values are represented above the bars.

Fig. S3| Whole chromosomal aberrations distinguish PanNEN subgroups. a. Hierarchical clustering of mean log2 ratios of chromosomal segments per autosome. Representative samples were analyzed for gains in chromosome 5 and 9 and loss in chromosome 11 (black boxes in the respective column annotations) using FISH. **b**. Quantification of chromosomal aberrations for chromosomes 5, 9 and 11 was performed by FISH. Where possible 40 cells per sample were counted. The distribution of signals per sample is depicted; each point represents the mean count of cells for the respective probe. Black dot represents the mean, error bars standard deviation. **c.** Distribution of absolute log2FC of pancreatic cell marker associated DMPs. Absolute log2FC ranges from 0.03 to 0.59 (x-axis), frequency of a given absolute log2FC on y-axis. **d.** Number of markers associated with each cell type represented in the bar plot; Markers associated with multiple cell types are also depicted; the cell type of the respective associations are linked (bottom annotation). **e.** Detailed image with sample names for main figure 3c. Methylation beta value of probes associated with IRX2 and PDX1. DMP probes of IRX2 and 10K probes associated with PDX1 (rows) for each sample. Lower panel depicts recurrently mutated genes.

Fig. S4| Pancreatic cell markers define PanNEN subgroups. IHC of ARX, PDX1 and SOX9 in Group A . All samples that underwent IHC for all three markers are shown. **b**. IHC analysis of ARX, PDX1 and SOX9 in Group B. The samples that underwent IHC for all three markers are shown. Scale bar: 20µm.

Fig. S5| Pancreatic cell atlas signatures define PanNEN subgroups. a. Stacked bar plot representing proportions of the pancreatic cell signatures; top annotation: PanNEN subgroup for the respective samples. **b.** Proportion of pancreatic cell signature in each PDAC sample (n=167). **c**. Proportion of pancreatic cell atlas signatures in NETG3 and NECs in all groups. Boxplots represent distribution of the proportion of atlas signature of α-, β-, ductal and acinar cells; Each

dot depicts the proportion of atlas signature of the respective cell type in a given sample. **d**. Proportion of pancreatic cell atlas signatures in *IRX2* hypo- / *PDX1* hypermethylated and *IRX2* hypo- / *PDX1* hypomethylated tumors of Group A. Boxplot represents distribution of the proportion of atlas signature of α-, β-, ductal and acinar cells (each main box) Group A tumors of respective *ARX* and *PDX1* methylation profile; Each dot depicts the proportion of atlas signature of the respective cell type in a given sample.

Fig. S6| Similarity of Group B and Exocrine cells based on DMPs distinguishing pancreatic cell types. Unsupervised class discovery using 10000 (10K) most variable methylation probes from DMPs distinguishing pancreatic cell types. Heatmap displays pairwise consensus values of the samples. Clusters are designated based on the positive correlation of pairwise consensus values between samples. Cluster 1 contains Group A, Group C and endocrine cells and Cluster 2 carries Group B and exocrine cells. **a.** Cumulative distribution function (CDF) curve of the resulting 'k-mer' count for 12-k's. Cluster count of 'two' and 'three' resulted in stable number of subgroups equally. **b**. Mean pairwise consensus values for each group in 2 clusters vs 3 clusters. **c.** Heatmap displaying methylation status of 10K variable probes in each of the newly identified clusters; Cluster 1 and 2. Color range blue to red represents methylation beta value, columns indicate samples and rows methylation probes. **d.** Distribution of absolute difference in mean methylation associated with the 10K probes between cluster 1 and cluster 2. Absolute difference in mean methylation ranges from 0.0001 to 0.5 (x-axis), the frequency of a given value is depicted in on y-axis. **e.** Boxplot representing the distribution of probes from the aforementioned 10K probes associated with promoters and gathered based on the gene it is localized to. Each dot represents the beta (methylation) value of the probe per group and the line depicts the median within a group (note: the median is only relevant when there are multiple probes within a given gene).

Fig S1

a

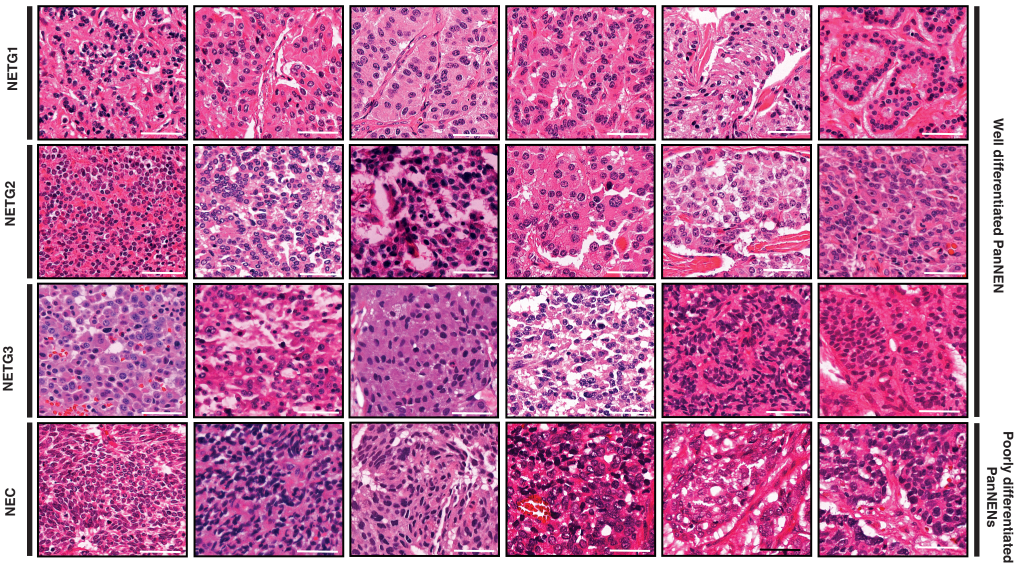

NETG1
NETG2
NETG3
NEC
Well differentiated PanNEN
Poorly differentiated PanNENs

b

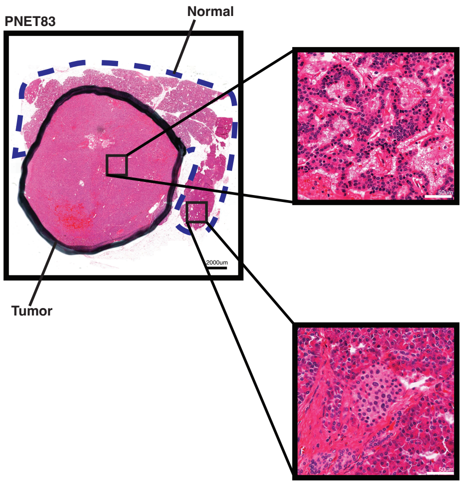

PNET83
Normal
Tumor
2000um
50um

c

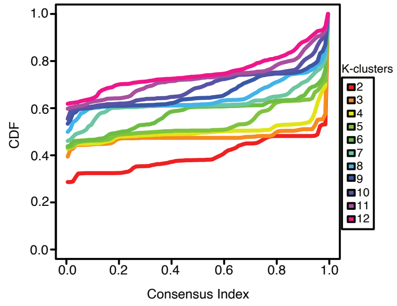

CDF
Consensus Index
K-clusters
2
3
4
5
6
7
8
9
10
11
12
0.0
0.2
0.4
0.6
0.8
1.0

d

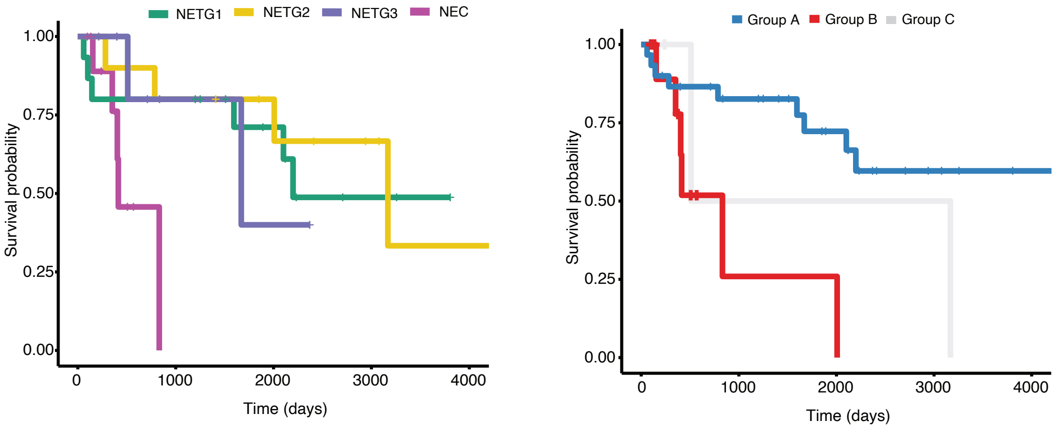

NETG1
NETG2
NETG3
NEC
Survival probability
Time (days)
1.00
0.75
0.50
0.25
0.00
0
1000
2000
3000
4000
Group A
Group B
Group C

Fig S2

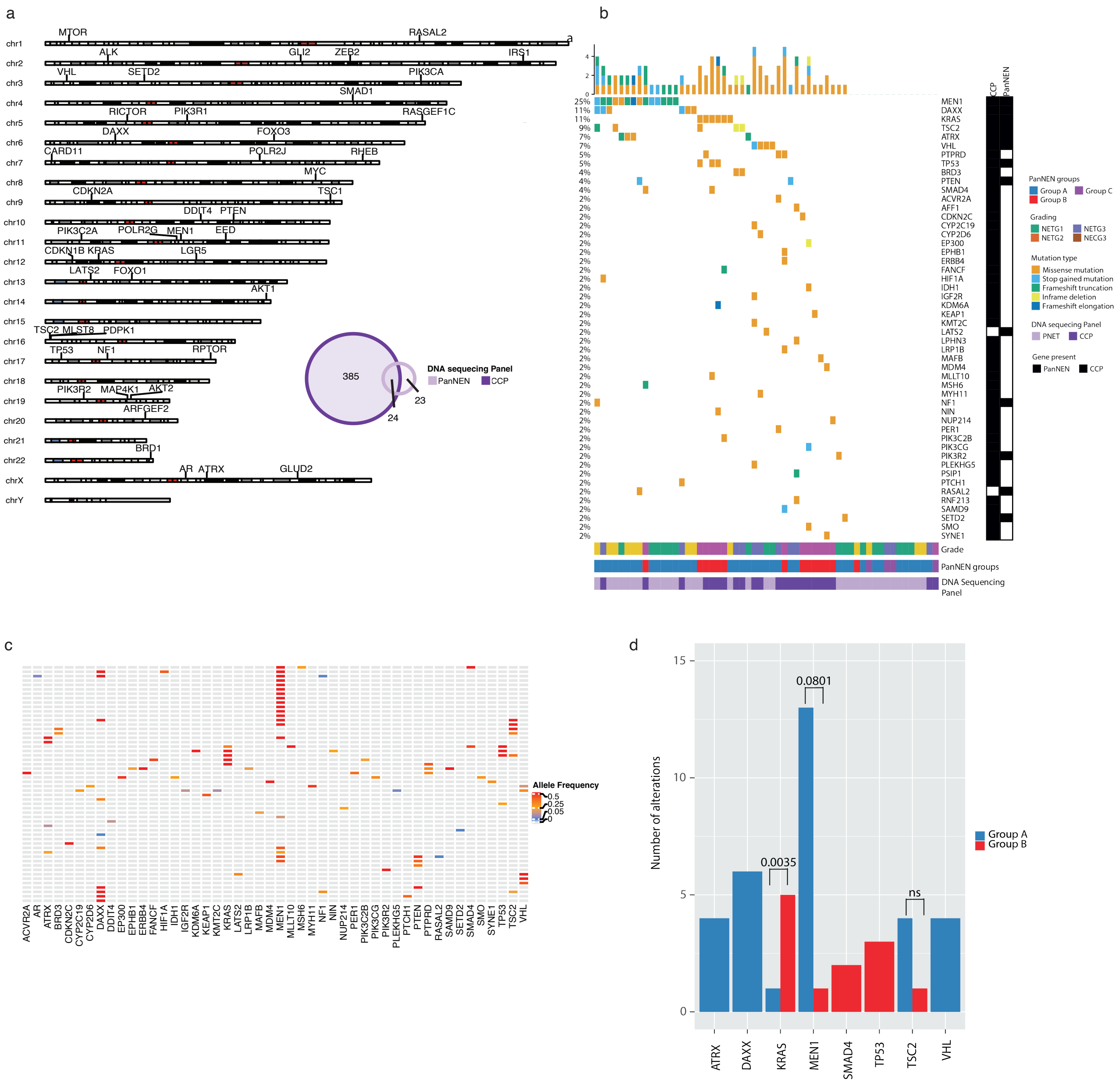

a
MTOR
RASAL2
ALK
GLI2
ZEB2
IRS1
VHL
SETD2
PIK3CA
SMAD1
RICTOR
PIK3R1
RASGEF1C
DAXX
FOXO3
CARD11
POLR2J
RHEB
MYC
CDKN2A
TSC1
DDIT4
PTEN
PIK3C2A
POLR2G
MEN1
EED
CDKN1B
KRAS
LGR5
LATS2
FOXO1
AKT1
TSC2
MLST8
PDPK1
TP53
NF1
RPTOR
PIK3R2
MAP4K1
AKT2
ARFGEF2
BRD1
AR
ATRX
GLUD2
chr1
chr2
chr3
chr4
chr5
chr6
chr7
chr8
chr9
chr10
chr11
chr12
chr13
chr14
chr15
chr16
chr17
chr18
chr19
chr20
chr21
chr22
chrX
chrY
385
23
24
DNA sequecing Panel
PanNEN
CCP
b
CCP
PanNEN
MEN1
DAXX
KRAS
TSC2
ATRX
VHL
PTPRD
TP53
BRD3
PTEN
SMAD4
ACVR2A
AFF1
CDKN2C
CYP2C19
CYP2D6
EP300
EPHB1
ERBB4
FANCF
HIF1A
IDH1
IGF2R
KDM6A
KEAP1
KMT2C
LATS2
LPHN3
LRP1B
MAFB
MDM4
MLLT10
MSH6
MYH11
NF1
NIN
NUP214
PER1
PIK3C2B
PIK3CG
PIK3R2
PLEKHG5
PSIP1
PTCH1
RASAL2
RNF213
SAMD9
SETD2
SMO
SYNE1
Grade
PanNEN groups
DNA Sequencing Panel
PanNEN groups
Group A
Group C
Group B
Grading
NETG1
NETG3
NETG2
NECG3
Mutation type
Missense mutation
Stop gained mutation
Frameshift truncation
Inframe deletion
Frameshift elongation
DNA sequecing Panel
PNET
CCP
Gene present
PanNEN
CCP
c
Allele Frequency
0.5
0.25
0.05
0
ACVR2A
AR
ATRX
BRD3
CDKN2C
CYP2C19
CYP2D6
DAXX
DDIT4
EP300
EPHB1
ERBB4
FANCF
HIF1A
IDH1
IGF2R
KDM6A
KEAP1
KMT2C
KRAS
LATS2
LRP1B
MAFB
MDM4
MEN1
MLLT10
MSH6
MYH11
NF1
NIN
NUP214
PER1
PIK3C2B
PIK3CG
PIK3R2
PLEKHG5
PTCH1
PTEN
PTPRD
RASAL2
SAMD9
SETD2
SMAD4
SMO
SYNE1
TP53
TSC2
VHL
d
Number of alterations
0.0801
0.0035
ns
Group A
Group B
ATRX
DAXX
KRAS
MEN1
SMAD4
TP53
TSC2
VHL

Fig S3

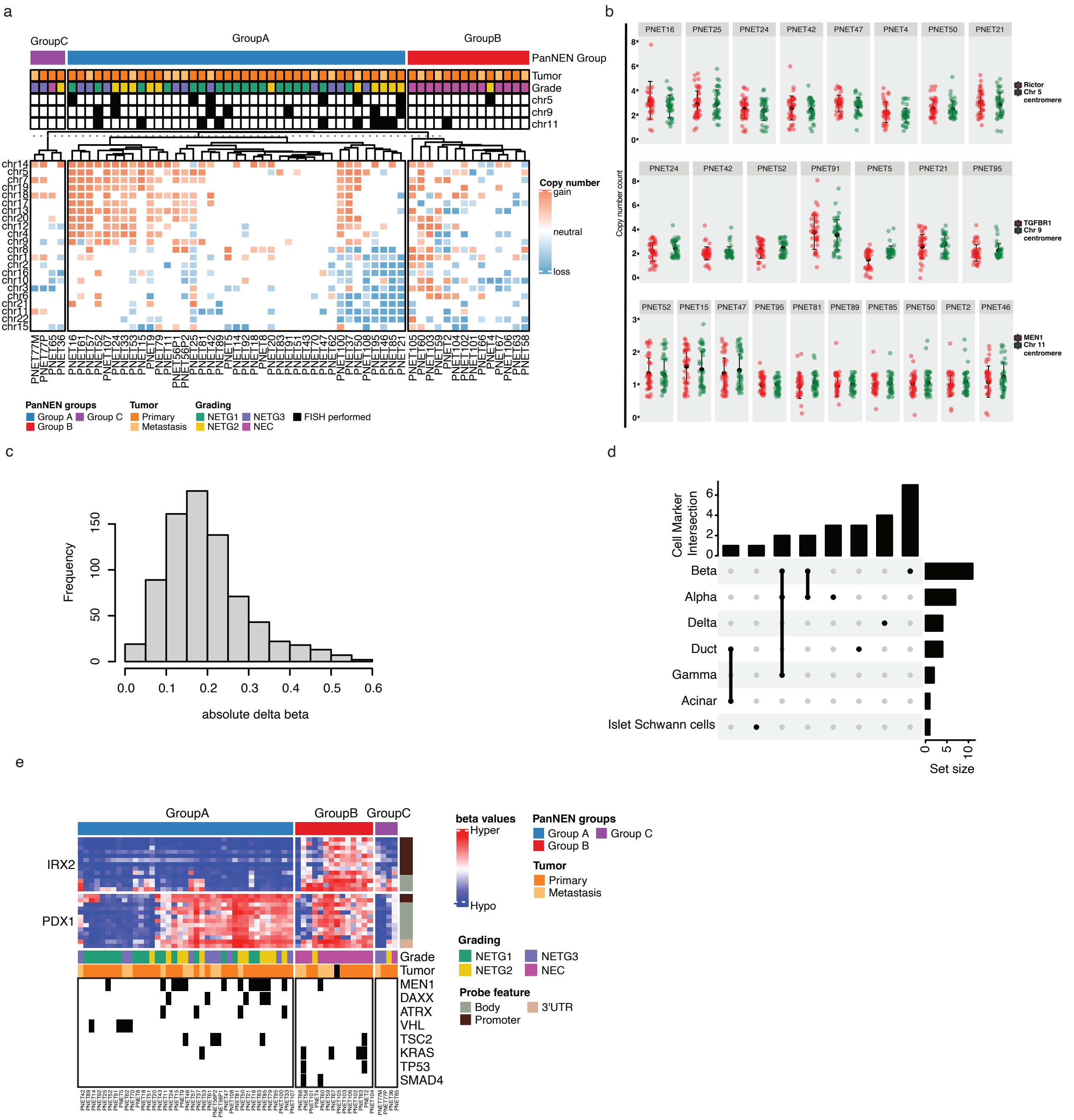
a
GroupC
GroupA
GroupB
PanNEN Group
Tumor
Grade
chr5
chr9
chr11
Copy number
gain
neutral
loss
PanNEN groups
Group A
Group C
Group B
Tumor
Primary
Metastasis
Grading
NETG1
NETG3
NETG2
NEC
FISH performed
b
Copy number count
Rictor
Chr 5 centromere
TGFBR1
Chr 9 centromere
MEN1
Chr 11 centromere
c
Frequency
absolute delta beta
d
Cell Marker Intersection
Beta
Alpha
Delta
Duct
Gamma
Acinar
Islet Schwann cells
Set size
e
GroupA
GroupB
GroupC
IRX2
PDX1
Grade
Tumor
MEN1
DAXX
ATRX
VHL
TSC2
KRAS
TP53
SMAD4
beta values
Hyper
Hypo
PanNEN groups
Group A
Group C
Group B
Tumor
Primary
Metastasis
Grading
NETG1
NETG3
NETG2
NEC
Probe feature
Body
3'UTR
Promoter

Fig S4

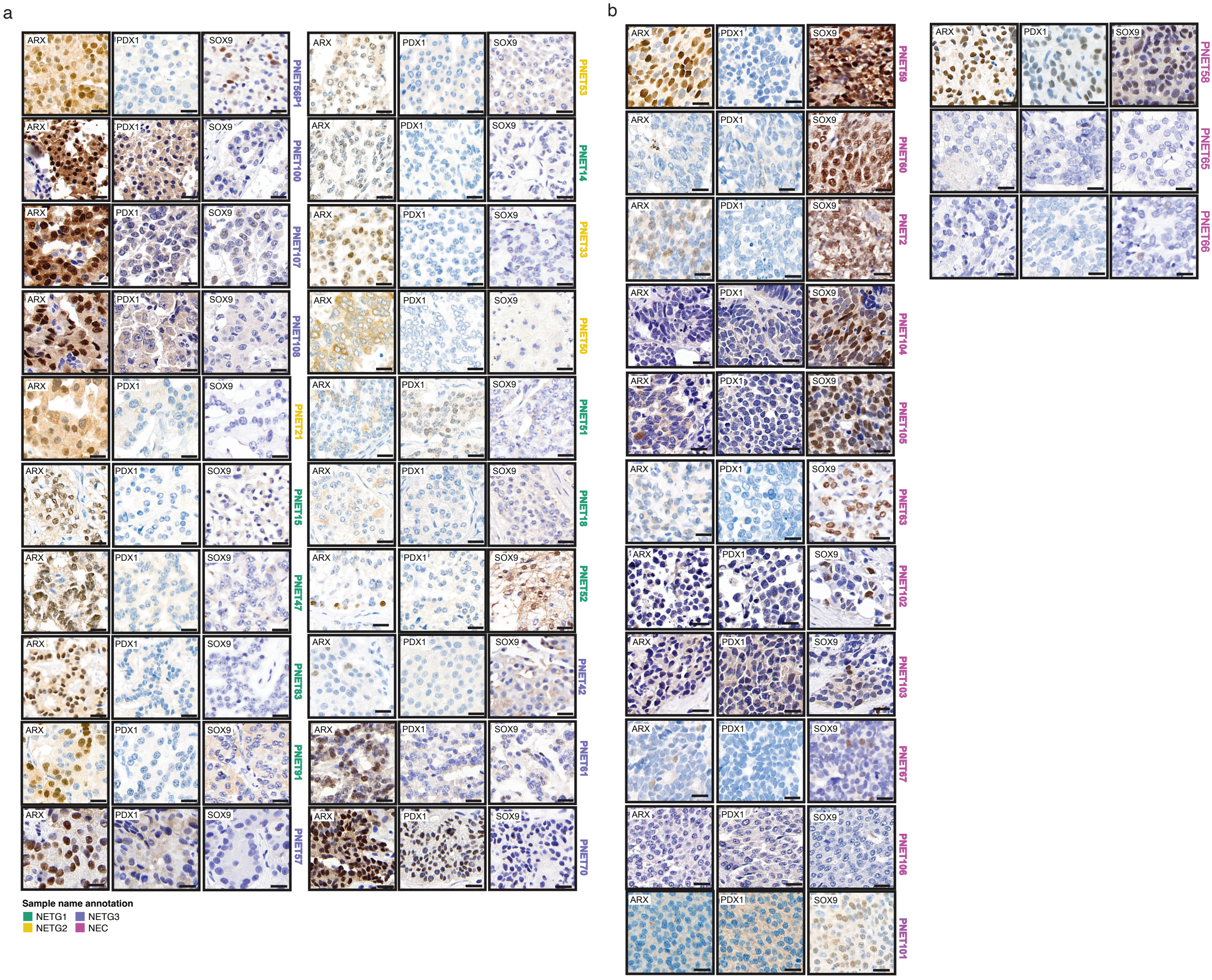
a
b
ARX
PDX1
SOX9
PNET56P1
PNET100
PNET107
PNET108
PNET21
PNET15
PNET47
PNET83
PNET91
PNET57
PNET53
PNET14
PNET33
PNET50
PNET51
PNET18
PNET52
PNET42
PNET61
PNET70
PNET59
PNET60
PNET2
PNET104
PNET105
PNET63
PNET102
PNET103
PNET67
PNET106
PNET101
PNET58
PNET65
PNET66
Sample name annotation
NETG1
NETG3
NETG2
NEC

Fig S5

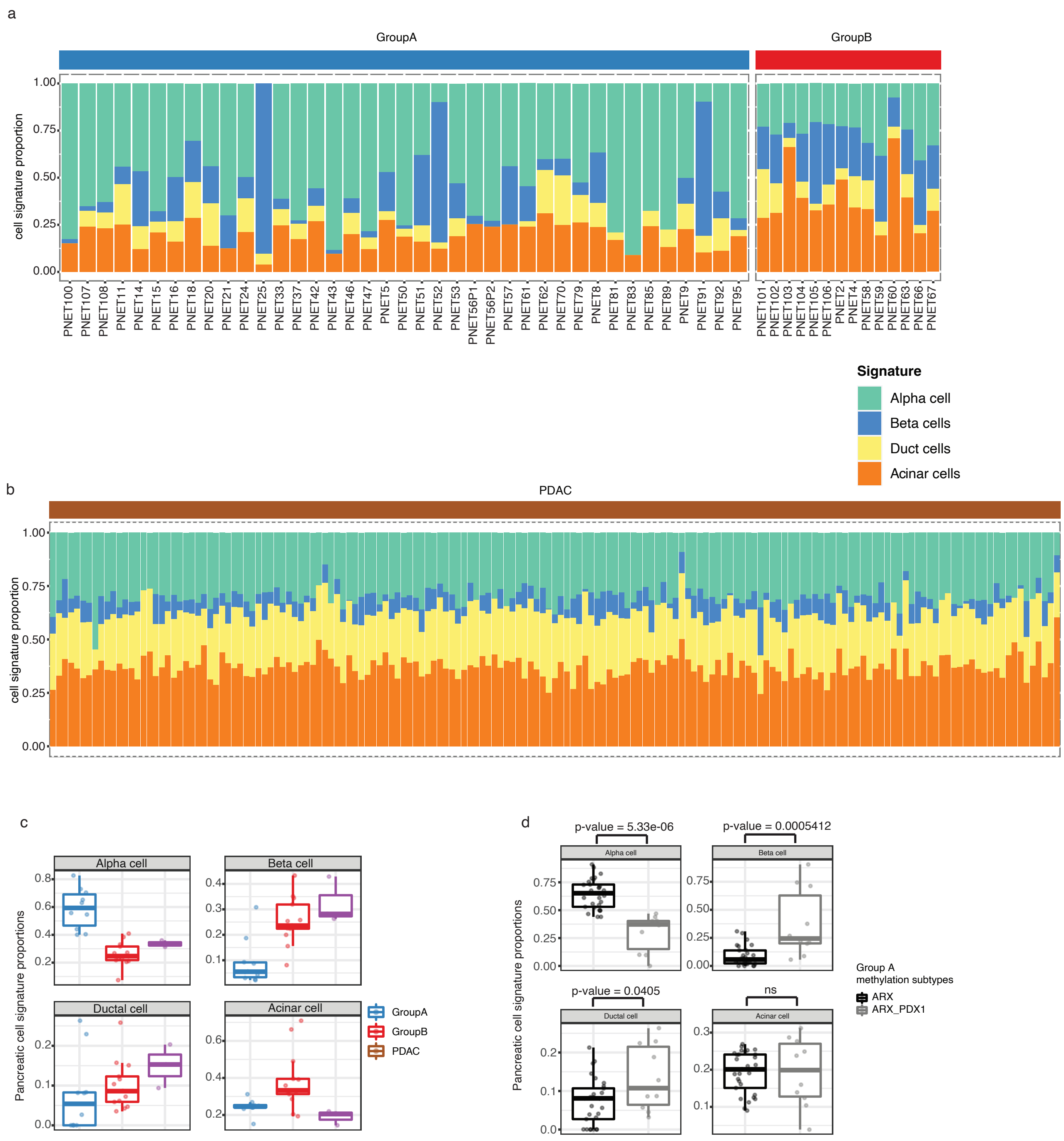
a
GroupA
GroupB
cell signature proportion
1.00
0.75
0.50
0.25
0.00
PNET100
PNET107
PNET108
PNET11
PNET14
PNET15
PNET16
PNET18
PNET20
PNET21
PNET24
PNET25
PNET33
PNET37
PNET42
PNET43
PNET46
PNET47
PNET5
PNET50
PNET51
PNET52
PNET53
PNET56P1
PNET56P2
PNET57
PNET61
PNET62
PNET70
PNET79
PNET8
PNET81
PNET83
PNET85
PNET89
PNET9
PNET91
PNET92
PNET95
PNET101
PNET102
PNET103
PNET104
PNET105
PNET106
PNET2
PNET4
PNET58
PNET59
PNET60
PNET63
PNET66
PNET67
Signature
Alpha cell
Beta cells
Duct cells
Acinar cells
b
PDAC
cell signature proportion
1.00
0.75
0.50
0.25
0.00
c
Pancreatic cell signature proportions
Alpha cell
0.8
0.6
0.4
0.2
Beta cell
0.4
0.3
0.2
0.1
Ductal cell
0.2
0.1
0.0
Acinar cell
0.6
0.4
0.2
GroupA
GroupB
PDAC
d
Pancreatic cell signature proportions
p-value = 5.33e-06
Alpha cell
0.75
0.50
0.25
0.00
p-value = 0.0005412
Beta cell
0.75
0.50
0.25
0.00
p-value = 0.0405
Ductal cell
0.2
0.1
0.0
ns
Acinar cell
0.3
0.2
0.1
Group A
methylation subtypes
ARX
ARX_PDX1

FIg S6

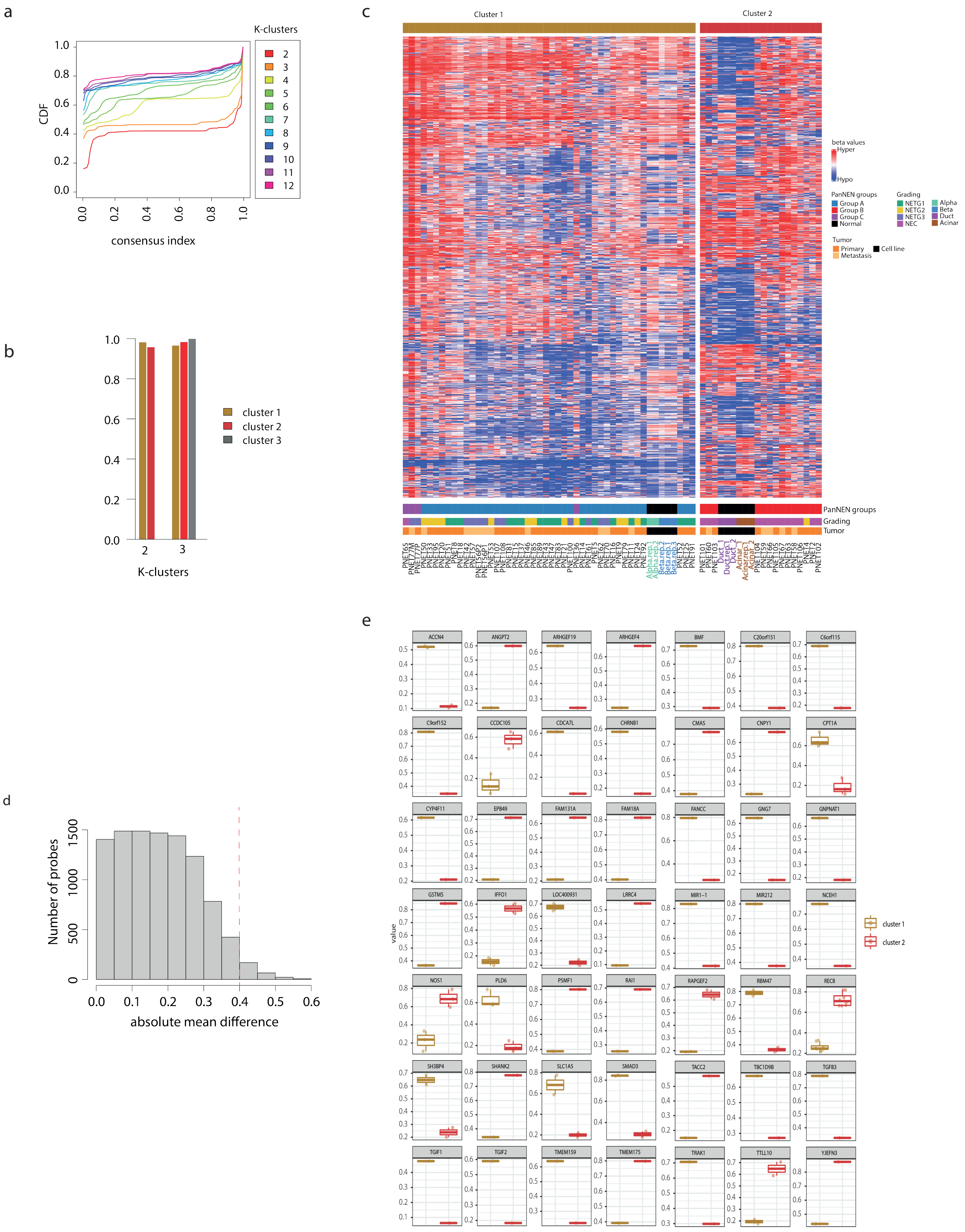
a
K-clusters
2
3
4
5
6
7
8
9
10
11
12
CDF
consensus index
b
K-clusters
cluster 1
cluster 2
cluster 3
c
Cluster 1
Cluster 2
beta values
Hyper
Hypo
PanNEN groups
Group A
Group B
Group C
Normal
Grading
NETG1
NETG2
NETG3
NEC
Alpha
Beta
Duct
Acinar
Tumor
Primary
Metastasis
Cell line
PanNEN groups
Grading
Tumor
d
Number of probes
absolute mean difference
e
value
cluster 1
cluster 2
ACCN4
ANGPT2
ARHGEF19
ARHGEF4
BMF
C20orf151
C6orf115
C9orf152
CCDC105
CDCA7L
CHRNB1
CMAS
CNPY1
CPT1A
CYP4F11
EPB49
FAM131A
FAM18A
FANCC
GNG7
GNPNAT1
GSTM5
IFFO1
LOC400931
LRRC4
MIR1−1
MIR212
NCEH1
NOS1
PLD6
PSMF1
RAI1
RAPGEF2
RBM47
REC8
SH3BP4
SHANK2
SLC1A5
SMAD3
TACC2
TBC1D9B
TGFB3
TGIF1
TGIF2
TMEM159
TMEM175
TRAK1
TTLL10
YJEFN3
